# The Use of Hyperimmune Chicken Reference Sera Is Not Appropriate for the Validation of Influenza Pseudotype Neutralization Assays

**DOI:** 10.3390/pathogens6040045

**Published:** 2017-09-25

**Authors:** Francesca Ferrara, Eleonora Molesti, Simon Scott, Giovanni Cattoli, Nigel Temperton

**Affiliations:** 1 Viral Pseudotype Unit, Medway School of Pharmacy, University of Kent, Chatham, Kent ME4 4TB, UK; ff63@kentforlife.net (F.F.); em296@kentforlife.net (E.M.); S.D.Scott@kent.ac.uk (S.S.); 2 Animal Production and Health Laboratory, Joint FAO/IAEA Division, International Atomic Energy Agency, A-2444 Seibersdorf, Austria; G.Cattoli@iaea.org

**Keywords:** influenza, serology, standards, validation, pseudotypes, neutralization

## Abstract

The pseudotype particle neutralization test (pp-NT) is a next-generation serological assay employed for the sensitive study of influenza antibody responses against hemagglutinin (HA), including stalk-directed antibodies. However, a validation of this assay has yet to be performed, and this limits its use to primarily research laboratories. To identify possible serological standards to be used in optimization and validation of the pp-NT, we have evaluated the cross-reactivity of hyperimmune chicken reference antisera in this assay. Our findings show that the cross-reactivity detected by the pp-NT is only partly explained by phylogenetic relationships and protein homology between the HA subtypes analysed; further studies are necessary to understand the origin of the cross-reactivity detected, and reference standards with higher specificity should be evaluated or generated *de novo* for future use in pp-NT.

## 1. Introduction

Serological methods such as single radial haemolysis (SRH), haemagglutination inhibition (HI) and microneutralization (MN) are cost-effective and widely-used methodologies to monitor the circulation and prevalence of influenza viruses, and are also employed in vaccine immunogenicity studies [1]. However, these assays have numerous shortcomings, especially related to the variability of the reagents, their standardization between laboratories, and their ability to detect haemagglutinin (HA) stalk-directed antibodies. Enzyme-linked immunosorbent assays (ELISAs) are used to detect stalk-directed antibodies, but are unable to differentiate between binding and neutralizing antibodies.

Recently, the use of replication-deficient viruses in neutralization assays has been widely evaluated as a potential alternative to MN assays. For example, HA pseudotype particles (pp) harbouring the HA embedded in the viral envelope and having a lentiviral vector as backbone have been used effectively for this purpose [2,3]. The pseudotype particle neutralization test (pp-NT) appears to be more sensitive than other functional assays in the detection of the antibody response directed against the HA stalk [4]. Consequently, pp could be used to effectively study heterosubtypic antibody responses directed against the HA stalk region. Importantly, pp-NTs have been shown to correlate strongly with other classical serological assays [5,6,7,8]. Since pp are replication-defective, they offer a safe alternative to wild-type virus methods (that require Biosafety Level 3 containment), and the detection of antibody responses is not influenced by the variability of blood-based reagents as observed in other assays (i.e., red blood cells in HI). Unfortunately, the validation of pp-NT, which will permit its more extensive use in surveillance as well as in pre-clinical and clinical studies, has not yet been established. The validation of pp-NT will allow the certification of its use in product release and stability control (if, in the future, HA stalk-directed monoclonal antibodies will be licensed) and in evaluating antibody responses elicited by current and “next-generation universal” influenza vaccines. 

The availability of appropriate reference materials is essential for the optimization and validation of an assay. Appropriate reference standards are especially useful when the specificity, sensitivity, precision, and accuracy of an assay are evaluated for the first time [9], but are also essential when other assay parameters (e.g., dilution range or calibration curves) are established [10]. Furthermore, reference standards have an essential role in monitoring assay stability and consistency over different analytical sessions (e.g., days) [11]. Reference materials are also useful when multisite validation of an assay is performed. For example, the use of reference standard sera has been shown to be extremely useful to increase consistency between laboratories using HI and SRH [12]. Chicken reference antisera against all the HA subtypes are commonly generated and used for influenza virus typing in the HI assay [13,14], and thus should be investigated as possible controls and reference materials in pp-NTs, which was the purpose of this study.

## 2. Results and Discussion

We have evaluated the neutralization activity and cross-reactivity of chicken reference antisera against a panel of pp bearing HAs of a representative strain (where possible of avian origin), for each HA subtype. Unfortunately, H6 and H13 influenza pp were not used in this study since appropriate pp bearing the HA of these two subtypes were not available. 

**Figure 1:**
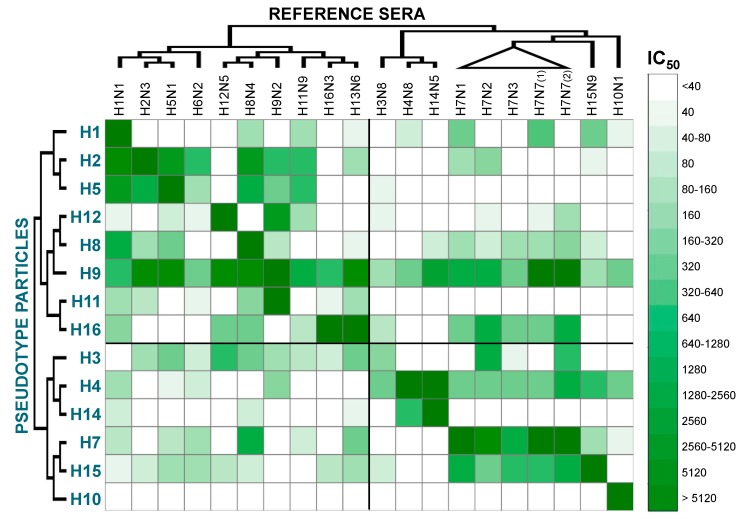
Cross-reactivity map of pseudotype particles (pp) and reference sera based on IC_50_. The IC_50_ values used for the cross-reactivity heat map were the result of arithmetic means between one or more different experiments performed when the same batch of sera and the same batch of pp were available, in order to minimize potential additional variability factors. Note: H7N7 (1) is A/turkey/England/647/1977 (H7N7) and H7N7 (2) is A/England/268/1996 (H7N7).

The neutralizing titres resulting after analysis with GraphPad Prism software were elaborated using Microsoft Excel and the statistical software R to generate a “heat map” (Figure 1). Phylogenetic analysis was also used to highlight relationships between HA subtypes. Observing Figure 1, some features of the cross-reactivity map become immediately discernible: in the top left, there are Group 1 HA pp against Group 1 reference antisera, in the bottom right there are Group 2 HA pp against Group 2 reference antisera, and in the bottom left and top right there are the results of pp tested with reference antisera from a different group. The cross-reactivity map diagonal represents pp that were tested against matching antisera, and as expected, the higher neutralization titres are usually found in this part of the table. It can also be noticed that two map-shifts are present, since the H6pp and H13pp are missing. A clear symmetry can be visualised using the diagonal as symmetry axis, and in general, high neutralization titres are found when pp are tested with antisera of the same group (top left and bottom right). Conversely, sometimes antisera neutralize pp that harbour HAs of a completely different subtype/group. Other important features are that pp were usually neutralized by antisera generated against closely related viruses: in fact, using the two phylogenetic trees as guides, it is possible to identify clusters of darker green in relation to phylogenetic clades; for example, the H2-H5, H12-H8-H9, H11-H16-H13, H4-H14, and H7-H15. The cross-reactivity heat map shows that influenza reference antisera are usually able to efficiently neutralize HA-matched pp, and minor variation in the neutralizing titres can be observed when the serum was generated against paired (same subtype but different strain) HAs. Additionally, cross-reactive responses can be detected not only when phylogenetic relationships are present between the HA of the pp tested and the HA used as antigens to generate the antisera, but also between HA and antisera that share less similarity (Figure 1). 

Looking at Figure 1, distinctive cross-reactivities can be observed. H1pp was highly neutralized by the matched antisera, but not by antisera generated against closely related HAs (H2/H5); however, it was neutralized by other Group 1 antisera (H8N4 and H11N9) and additionally by three Group 2 antisera. The H2pp and H5pp were neutralized by each other’s antisera, by the antisera generated against the most closely related HAs (H1 and H6), but also by H8N4, H9N2, and H11N9 antisera. Even though closely related, H8pp and H12pp did not exhibit neutralization by each other’s antisera; however, they were neutralized by the antisera generated against the closely related H9N2. Furthermore, H8pp was also neutralized by the antisera generated against H1-H2-H5, and by some antisera of Group 2, especially the ones generated against H7 viruses. All the antisera, without distinction of HA subtype, neutralized H9pp: higher neutralization activities were detected for Group 1 antisera, for H14N5 and H7 antisera, especially anti-H7N7. H11pp and H16pp were neutralized by their matched antisera and by antisera generated against other closely related HA, and by H1N1 antisera. For H16pp, neutralization activity was also detected when H12N5, H8N4, and H7 antisera were used. For Group 2 pp, A/Udorn/207/1972 H3pp (a human isolate) was poorly neutralized by the matched avian antisera, but was neutralized by the anti-H7N2 sera, one of the H7N7 antisera, and by numerous Group 1 antisera. H4pp was neutralized by all Group 2 antisera and by the H9N2 antisera. H14pp was neutralized by the matching antisera and by the antisera generated against the closely related H4. H7pp and H15pp were neutralized by their matched antisera and by each other’s antisera; Group 1 sera were also found to cross-react with H15pp and some (H6N2 and H13N9) also with H7pp. H10pp was only neutralized by its matching antisera and not by any others.

Many studies have previously observed that chicken antisera generated using whole virus present a lower specificity in HI and/or immunodot-blot assays in comparison to the ones generated through HA-expressing DNA vaccination or recombinant HA1 vaccination [15,16]. However, since a certain level of cross-reactivity is also observed with DNA or recombinant protein vaccination, cross-reactive HA-directed antibodies are likely involved [16]. 

**Figure 2:**
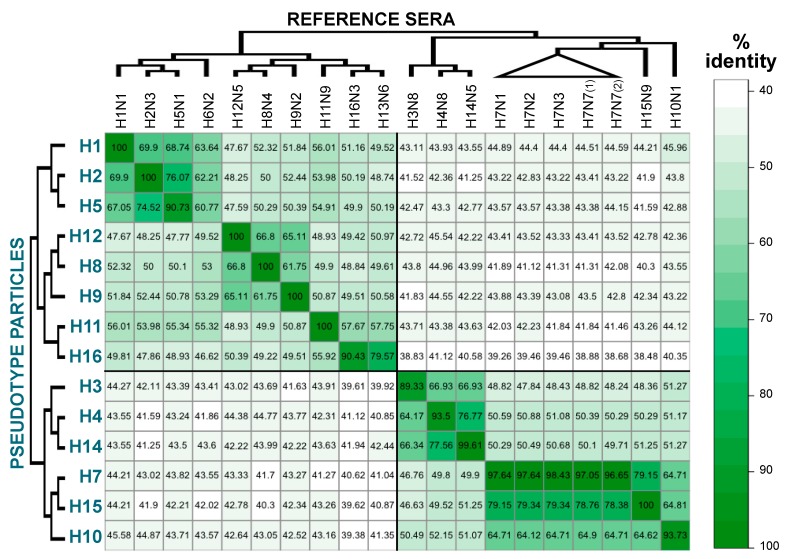
Cross-reactivity map of pp and reference sera based on percentage of amino acid identity. Percentage identities between haemagglutinin (HA) amino acid sequences trimmed of their N-terminal signal, the transmembrane region, and the cytoplasmic tail were calculated by pair-wise alignments using Jalview and coloured using the heatmap function in R. In the figure, H7N7 (1) is A/turkey/England/647/1977 (H7N7) and H7N7 (2) is A/England/268/1996 (H7N7).

In this study, commonly used reference antisera (for haemagglutination inhibition assay, agar gel immunodiffusion test, and agar gel precipitation test) show high neutralizing responses and cross-reactivity between different strains/subtypes. This could be problematic not only for HI typing, but especially for pp-NT. In fact, in the dilution range analysed, the pp-NT could not discriminate between two distinct pp (e.g., H16 pp was neutralized at the same dilution tested by both H16N3 and H13N6 sera). Preparation of the standard material through dilution of the commonly used reference standard or the use of monoclonal antibody mixtures (with or without the presence of a serum matrix) showing high specificity could be more effective approaches to establish reference materials to be used to validate, standardise, and control pp-NTs. To better understand which factors are underlying the observed cross-reactivity, we have evaluated the percentage amino acid identity between the HAs present on the pp and the HAs used for antisera generation. A percentage-identity heat map was then produced (Figure 2). 

This map shows a similar overall pattern to the cross-reactivity map (Figure 1). There is a symmetry following the diagonal, the darker green is concentrated in the top left and bottom right sectors of the map. However, compared to the previous map (Figure 1), dark green cannot be observed in the two other sectors of the maps; thus, as expected, the similarity between strains belonging to different groups is low. Since it is difficult to highlight all the differences and similarities by eye, statistical analysis was performed to see whether any concordance or association between the maps was present. Kendall τ test shows that there is low association between percentage identity and neutralization titres (τ = 0.225, *p* = 2.38 × 10^−7^). This means that the percentage amino acid identity is a good approach to evaluate the cross-reactivity response, but other approaches should be used to understand and explain the cross-reactivity detected. Antigenic cartography has recently been used to evaluate the antigenic evolution/drift of different influenza viruses and to help vaccine strain selection [17,18]. This methodology could be used to analyse the data presented here, and should permit a representation of the antigenic interplay between different pp. In the absence of appropriate controls and the presence of high cross-reactivity responses, it will be difficult to assess the specificity of the pp-NT assay. Other parameters should be evaluated to understand if factors unrelated with the sera antibody content could interfere with the pp-NT assay. For example, the presence of virus-attachment inhibitors in the sera and serum treatments (e.g., heat-inactivation, pre-treatment with receptor-destroying enzymes) and/or the presence and type of adjuvant used can be assessed to optimise pp-NT assay conditions and to reduce non-specific neutralization if present. Additionally, the evaluation of possible haemolysis or other contaminants (e.g., lipids) of the serum samples is a factor that needs to be taken into consideration when the assay is optimised and validated [9]. 

## 3. Materials and Methods 

### 3.1. Reference Sera

The avian reference hyperimmune sera used for these studies and associated HI titres were provided by Dr. Giovanni Cattoli when he was at the Istituto Zooprofilattico delle Venezie (IZSVe), World Organization for Animal Health (OIE), Legnaro, Padua, Italy, and are reported in Table 1.

Reference avian sera against H5 and H7 influenza strains were provided by the Animal and Plant Health Agency (APHA, previously Animal Health and Veterinary Laboratories Agency) and are reported in Table 2.

Sera were produced by inoculation of specific pathogen-free chickens with whole influenza viruses, in accordance with OIE guidelines (World Organization for Animal Health). If necessary, sera were pre-adsorbed with chicken red blood cells (RBCs). Other pre-treatments (e.g., decomplementation at 56 °C for 30 min, treatment with receptor-destroying enzymes) were not performed.

### 3.2. Pseudotype Particles and Pseudotype Particle Neutralization Assays

Lentiviral pseudotyped particles were produced as described previously [8,19]. Briefly, the HIV gag-pol plasmid p8.91, the firefly luciferase-expressing plasmid pCSFLW, the HA-expressing plasmid (Table 3), and the pCAGGS-TMPRSS2 or HAT were co-transfected into human embryonic kidney HEK293T/17 cells using Polyethylenimine (PEI). After 24 h incubation, 1 U of recombinant neuraminidase from *Clostridium perfringens* (Sigma) was added to facilitate pseudotype exit from the producer cells. Supernatant was harvested forty-eight hours post-transfection, filtered through 0.45 μm filters, and stored at −80 °C. 

The pp-NTs were performed as described elsewhere [19], using 5 µL of each serum sample (starting dilution 1:40) and using a pp input of 1 × 10^6^ RLU/well. IC_50_ neutralization titres were calculated using GraphPad Prism^®^ expressed as dilution factor; then for further statistical analysis, they were categorised into 17 groups according to the dilution tested and as reported in Table 4.

A cross-reactivity map (pp versus reference antisera) completed using the neutralization groups for further statistical analysis, was designed in a Microsoft^®^ Excel 2011 spread sheet and then saved as a comma-separated values (csv) file.

### 3.3. Bioinformatic Analysis

Percentage identity between HA amino acid sequences of pp and reference sera antigens were calculated to check if cross-reactivity could be explained by overall sequence similarity. Amino acid sequences of the HA used in neutralization assays, and used to generate the reference antisera, were downloaded from the Influenza Virus Resource, the Influenza Research database, and the Global Initiative on Sharing Avian Influenza Data (GISAID) EpiFlu™ platform [20]. The accession numbers of the HAs used in pp-NT assays are reported in Table 3; HA accession numbers of the reference antisera are reported in Table 1 and Table 2. For A/mallard/Italy/3817-34/2005 (H9N2), the HA sequence was not available at the time of analysis, and therefore the pp sequence was used as a reference. Additionally, some of the HA sequences in the databases were incomplete, which complicates the analysis. To minimize this, it was decided to evaluate the percentage identity only for the amino acids that constitute the extracellular part of the HA (amino acids from 24 to 547 using H3 numbering), which were available for all HAs used. All sequences were aligned using MUSCLE algorithm [21] and Jalview software [22]. Subsequently, the sequences were trimmed of their N-terminal signal sequence, the transmembrane region, and the cytoplasmic tail. Percentage identities between amino acid sequences were calculated by pair-wise alignments using Jalview before being reported in a Microsoft^®^ Excel 2011 spreadsheet and saved as a csv file. The phylogenetic trees shown alongside the cross-reactivity and the percentage identity tables were generated using Molecular Evolutionary Genetics Analysis (MEGA) software [23]: the aligned sequences were imported and trees derived using Unweighted Pair Group Method with Arithmetic mean (UPGMA), the simplest method of tree construction based on pairwise evolutionary distances. The trees generated were manually modified using MEGA and FigTree (http://tree.bio.ed.ac.uk/software/figtree/).

### 3.4. Statistical Analysis

Cross-reactivity tables for the IC_50_ neutralization titres, expressed as group, and for percentage amino acid identity, were completed using Microsoft^®^ Excel 2011. R statistical software was then used to analyse the data and design a “heat-map” which colour codes the neutralization titres and the percentage identity. These codes are based on the use of the software package “RColorBrewer”(which permits a personalised colour palette to be built), and “gplots”, a package that contains functions for the graphical interface. The “heatmap.2” function was eventually used to assign a colour to each IC_50_ group or percentage identity value. Kendall τ (tau) statistics (“Kendall” package) was also run using R software to check if association/correlation between measured IC_50_ titres and percentage identity was present.

## 4. Conclusions

To conclude, the results presented here show that the high sensitivity and the propensity of the pp-NT assay to detect cross-reactive responses does not permit the use of current chicken reference standard antisera as reference materials to validate the assay. Until more appropriate standards (e.g., ferret sera or monoclonal antibody mixtures) are developed to further progress optimisation and validation of the pp-NT assay, the routinely used reference standards should be used as positive neutralization controls only in experimental research settings.

## Figures and Tables

**Table 1:** IZSVe avian influenza reference antisera for haemagglutination inhibition assay, agar gel immunodiffusion test, and agar gel precipitation test.

Antigen Strain Name	Subtype	HA Accession Number	HI Titre
A/duck/Italy/1447/2005	H1N1	HF563054.1	1:512
A/duck/Germany/1215/1973	H2N3	CY014710.1	1:512
A/psittacine/Italy/2873/2000	H3N8	GQ247846.1 *	1:256
A/cockatoo/England/1972	H4N8	GQ247847.1 *	1:128
A/turkey/Canada/1965	H6N2	GQ247851.1 *	1:256
A/turkey/Ontario/6118/1968	H8N4	CY014659.1	1:512
A/mallard/Italy/3817-34/2005	H9N2	Not Applicable	1:256
A/ostrich/South Africa/2001	H10N1	GQ247860.1 *	1:512
A/duck/Memphis/546/1974	H11N9	AB292779.1	1:1024
A/duck/Alberta/60/1976	H12N5	CY130078.1	1:128
A/gull/Maryland/704/1977	H13N6	D90308.1	1:1024
A/mallard/Gurjev/263/1982	H14N5	M35997	1:512
A/shearwater/Australia/2576/1979 **	H15N9	CY130102.1	1:2048
A/gull/Denmark/68110/2002	H16N3	GQ247872.1 *	1:256

* Partial sequence; ** Also known as A/shearwater/West Australia/2576/1979.

**Table 2:** Animal and Plant Health Agency (APHA) avian influenza reference antisera.

Antigen Strain Name	Subtype	HA Accession Number	HI Titre
A/chicken/Scotland/1959	H5N1	CY015081.1	Not available
A/African starling/England/983/1979	H7N1	AF202232.1	Not available
A/chicken/Wales/1306/2007	H7N2	EF675618.1	Not available
A/chicken/England/4054/2006	H7N3	EF467826.1	Not available
A/England/268/1996	H7N7	AF028020.1	Not available
A/turkey/England/647/1977	H7N7	AF202247.1	Not available

**Table 3:** Haemagglutinin-encoding plasmids used for pseudotype production.

Plasmid Backbone	HA	HA Accession Number
pI.18	A/duck/Italy/1447/2005 H1	HF563054.1
pI.18	A/Korea/426/1968 H2	CY125846.1
phCMV1	A/duck/Germany/1215/1973 H2	CY014710.1
pI.18	A/Udorn/307/1972 H3	DQ508929.1
phCMV1	A/duck/Czechoslovakia/1956 H4	D90302.1
pI.18	A/Vietnam/1194/2004 H5	ABP51976.1
pI.18	A/chicken/Italy/1082/1999 H7	ABR37396.1
phCMV1	A/turkey/Ontario/6118/1968 H8	CY014659.1
pI.18	A/Hong Kong/1073/1999 H9	AJ404626.1
phCMV1	A/chicken/Germany/N49 H10	CY014671.1
phCMV1	A/duck/Memphis/546/1974 H11	AB292779.1
phCMV1	A/duck/Alberta/60/1976 H12	CY130078.1
phCMV1	A/gull/Maryland/704/1977 H13	D90308.1
phCMV1	A/mallard/Astrakhan/263/1982 H14	AB289335.1
phCMV1	A/shearwater/West Australia/2576/1979 H15	CY130102.1
phCMV1	A/black-headed gull/Sweden/2/1999 H16	AY684888.1

**Table 4:** Categories of IC_50_ values used for statistics.

Group	IC_50_ Values	Dilution Factor
0	<35	<40
1	35–45	40
2	45–75	40–80
3	75–85	80
4	85–150	80–160
5	150–170	160
6	170–310	160–320
7	310–330	320
8	330–630	320–640
9	630–670	640
10	670–1270	640–1280
11	1270–1290	1280
12	1290–2550	1280–2560
13	2550–2570	2560
14	2570–5100	2560–5120
15	5100–5140	5120
16	>5140	>5120

## References

[1] Trombetta C., Perini D., Mather S., Temperton N., Montomoli E. (2014). Overview of Serological Techniques for Influenza Vaccine Evaluation: Past, Present and Future. Vaccines.

[2] Carnell G.W., Ferrara F., Grehan K., Thompson C.P., Temperton N.J. (2015). Pseudotype-based neutralization assays for influenza: A systematic analysis. Front. Immunol..

[3] Ferrara F., Molesti E., Temperton N. (2015). The application of pseudotypes to influenza pandemic preparedness. Future Virol..

[4] Corti D., Voss J., Gamblin S.J., Codoni G., Macagno A., Jarrossay D., Vachieri S.G., Pinna D., Minola A., Vanzetta F. (2011). A neutralizing antibody selected from plasma cells that binds to group 1 and group 2 influenza A hemagglutinins. Science.

[5] Garcia J.-M., Lagarde N., Ma E.S.K., de Jong M.D., Peiris J.S.M. (2010). Optimization and evaluation of an influenza A (H5) pseudotyped lentiviral particle-based serological assay. J. Clin. Virol..

[6] Wang W., Xie H., Ye Z., Vassell R., Weiss C.D. (2010). Characterization of lentiviral pseudotypes with influenza H5N1 hemagglutinin and their performance in neutralization assays. J. Virol. Methods.

[7] Alberini I., Del Tordello E., Fasolo A., Temperton N.J., Galli G., Gentile C., Montomoli E., Hilbert A.K., Banzhoff A., Del Giudice G. (2009). Pseudoparticle neutralization is a reliable assay to measure immunity and cross-reactivity to H5N1 influenza viruses. Vaccine.

[8] Temperton N.J., Hoschler K., Major D., Nicolson C., Manvell R., Hien V.M., Ha do Q., de Jong M., Zambon M., Takeuchi Y. (2007). A sensitive retroviral pseudotype assay for influenza H5N1-neutralizing antibodies. Influenza Respir. Viruses.

[9] Jacobson R.H. (1998). Validation of serological assays for diagnosis of infectious diseases. Rev. Sci. Tech..

[B10-pathogens-06-00045] 10.The United States Pharmacopeial Convention. ＜1033＞ Biological Assay Validation. 2010.

[11] Gray J.J., Wreghitt T.G., McKee T.A., McIntyre P., Roth C.E., Smith D.J., Sutehall G., Higgins G., Geraghty R., Whetstone R. (1995). Internal quality assurance in a clinical virology laboratory. II. Internal quality control. J. Clin. Pathol..

[12] Wood J.M., Gaines-Das R.E., Taylor J., Chakraverty P. (1994). Comparison of influenza serological techniques by international collaborative study. Vaccine.

[13] World Organization for Animal Health (2012). Avian Influenza.

[14] Dwyer D.E., Smith D.W., Catton M.G., Barr I.G. (2006). Laboratory diagnosis of human seasonal and pandemic influenza virus infection. Med. J. Aust..

[15] Shahsavandi S., Salmanian A.-H., Ghorashi S.A., Masoudi S., Fotouhi F., Ebrahimi M.M. (2011). Specific subtyping of influenza A virus using a recombinant hemagglutinin protein expressed in baculovirus. Mol. Biol. Rep..

[16] Lee C.-W., Senne D.A., Suarez D.L. (2006). Development and application of reference antisera against 15 hemagglutinin subtypes of influenza virus by DNA vaccination of chickens. Clin. Vaccine Immunol..

[17] Fouchier R.A.M., Smith D.J. (2010). Use of antigenic cartography in vaccine seed strain selection. Avian Dis..

[18] De Jong J.C., Smith D.J., Lapedes A.S., Donatelli I., Campitelli L., Barigazzi G., van Reeth K., Jones T.C., Rimmelzwaan G.F., Osterhaus A.D.M.E. (2007). Antigenic and Genetic Evolution of Swine Influenza A (H3N2) Viruses in Europe. J. Virol..

[19] Ferrara F., Molesti E., Böttcher-Friebertshäuser E., Cattoli G., Corti D., Scott S.D., Temperton N.J. (2012). The human Transmembrane Protease Serine 2 is necessary for the production of Group 2 influenza A virus pseudotypes. J. Mol. Genet. Med..

[20] (2017). GISAID: Global initiative on sharing all influenza data—From vision to reality. Euro Surveill..

[21] Edgar R.C. (2004). MUSCLE: Multiple sequence alignment with high accuracy and high throughput. Nucleic Acids Res..

[22] Waterhouse A.M., Procter J.B., Martin D.M.A., Clamp M., Barton G.J. (2009). Jalview Version 2—A multiple sequence alignment editor and analysis workbench. Bioinformatics.

[23] Tamura K., Peterson D., Peterson N., Stecher G., Nei M., Kumar S. (2011). MEGA5: Molecular evolutionary genetics analysis using maximum likelihood, evolutionary distance, and maximum parsimony methods. Mol. Biol. Evol..

